# Demineralization of Enamel in Primary Second Molars Related to Properties of the Enamel

**DOI:** 10.1100/2012/587254

**Published:** 2012-05-02

**Authors:** N. Sabel, A. Robertson, S. Nietzsche, J. G. Norén

**Affiliations:** ^1^Department of Pedodontics, Institute of Odontology at the Sahlgrenska Academy, University of Gothenburg, Box 450, SE-405 30 Göteborg, Sweden; ^2^Department of Pediatric Dentistry, Public Dental Service, Odontologen, Box 7162, SE-402 33 Göteborg, Sweden; ^3^Centre of Electron Microscopy, Friedrich-Schiller-University, Bachstr. 18, DE- 07743 Jena, Germany

## Abstract

Enamel structure is of importance in demineralization. Differences in porosity in enamel effect the rate of demineralization, seen between permanent and deciduous teeth. Individual differences have been shown in the mean mineral concentration values in enamel, the role of this in demineralization is not thoroughly investigated. The aim of this study was to study variations of depths of artificial lesions of demineralization and to analyze the depth in relation to variations in the chemical and mineral composition of the enamel. A demineralized lesion was created in second primary molars from 18 individuals. Depths of lesions were then related to individual chemical content of the enamel. Enamel responded to demineralization with different lesion depths and this was correlated to the chemical composition. The carbon content in sound enamel was shown to be higher where lesions developed deeper. The lesion was deeper when the degree of porosity of the enamel was higher.

## 1. Introduction

Important factors in caries development and demineralization are the enamel structure and its chemical properties. When exposing enamel with a weak acid, a demineralized lesion emerges. The lesions may vary in depth, depending on the properties of the enamel of the tooth. The solubility of enamel to acid solutions is a function of the chemical content and degree of porosity in the tissue [1]. This is clearly seen when comparing enamel of primary and permanent teeth. Differences in the morphological structures exist between permanent and primary enamel. The degree of porosity in primary enamel explains the differences in demineralization and the tendency to dissolution in primary enamel compared with permanent teeth [2, 3]. The higher degree of porosity leads to an increase in permeability in the enamel and is caused by a higher interprismatic fraction (interprismatic area related to intraprismatic area) [2, 4]. How large impact the differences of the degree of porosity in the enamel have in demineralization in vivo is not yet known. 

The chemical content and mineralization of enamel are known to vary between different teeth [5–8]. The degree of mineralization and chemical content of enamel seem to be of importance for the diffusion rate between primary and permanent teeth [2, 9, 10]. It is not known if there is quantitative difference in the chemical composition in approximal enamel compared with buccal enamel, further there are no studies whether there are any differences within primary enamel of importance to demineralization. Yet to investigate is if the differences seen in the chemical content are of importance to rate of demineralization. Additionally, whether the degree of mineralization is correlated to the depth of a lesion caused by demineralization.

The aim was to study variations of depths of artificial lesions of demineralization and to analyze the depth in relation to variations in chemical composition and the degree of mineralization of the enamel of primary teeth.

The hypothesis of the study was that lesions would develop differently, depending on minor differences in chemical composition of the enamel.

## 2. Materials and Methods

### 2.1. Selection of Patients

The subjects were comprised of patients referred to the Clinic of Pediatric Dentistry, Public Dental Care, Västra Götaland, Sweden, during the first quarter of the year 2008, for extraction of a second primary molar for odontological reasons. Eighteen children became eligible for the study and 18 primary second molars were collected. The age of the patients varied from 3 to 16.5 years.

### 2.2. Experimental Design

Immediately after extraction, the teeth were put in a saline solution for one day, then stored in 70% ethanol prior to further experimental procedures.

The teeth were covered with an acid resistant nail varnish (Boots No 7 Colour Lock, Boots, England), leaving a window on the buccal surface unprotected. To create a demineralized lesion, the teeth were covered with one batch of 8% methyl cellulose gel and placed individually in a 30 mL tin, filled with one batch of demineralizing solution (0.1 mol/l lactic acid, pH 5.3), at a temperature of 37°C for 30 days [11].

After exposure to the demineralizing solution, the teeth were thoroughly rinsed in deionized water and macrophotos of the exposed surface were taken. The enamel surface was visually inspected as a qualitative analysis concerning color and structure.

Sagittal buccolingual sections, with a thickness of 100 *μ*m, were prepared in a Leitz low speed saw microtome (Leitz, Wetzlar, Germany). Since the sections of the teeth were brittle, a drop of super glue (Loctite, Henkel AG & Co. KGaA, Munich, Germany) and a cover glass were placed on the tooth before sawing for each section, preventing enamel breakage. One of the samples broke during transportation, leaving 17 sections to analyze.

### 2.3. Polarized Light Microscopy (POLMI)

All sections were examined dry in air in an Olympus polarizing light microscope (Olympus, Tokyo, Japan) using a *λ*-filter. The sections and depth of lesions were analyzed in Leica Application Suite (Leica Microsystems AG, Heerbrugg, Switzerland).

The depths of lesions were measured at three locations, measured from the surface of enamel to the bottom border of the structural change of enamel prisms. The mean of depth was calculated.

### 2.4. Microradiography

Contact microradiographs were made using an X-ray tube (type OEG-50 Machlett) with a Cu target and a focal spot of 1 mm, generating a polyenergetic spectrum. The tube focus was fixed at 242 mm from the recording surface of the specimen. High-definition photo plates (HTA photomask, San Jose, CA, USA) were exposed, with the specimen placed directly on the emulsion, to nickel filtered copper radiation programmed at 20 kV and 20 mA for 75 minutes. The plates were developed according to the manufacturer's instructions. The microradiographs were examined in low magnification with a 4x lens in an Olympus light microscope (Olympus, Tokyo, Japan) and further analyzed with ImageJ (Research Services Branch, National Institute of Mental Health, Bethesda, Maryland, USA).

The lesion depth was measured in a Leica Application Suite (Leica Microsystems AG, Heerbrugg, Switzerland), from the enamel surface to the bottom of the lesion, as a visible transition of radio lucency to radio opacity. The radio lucency of the sound enamel in each microradiograph was analyzed with *ImageJ* (Research Services Branch, National Institute of Mental Health, Bethesda, MD, USA), calculating the gray value. The relative decrease of gray value (Δgv) was calculated as the difference in gray value in the lesion compared to the gray value of the sound enamel in each microradiograph. The illumination in the microscope was ascertained by measuring the grayscale values over a digital image without any sample.

The depths of the lesions were estimated at three locations, measured from the surface of enamel to the bottom border of the structural change of enamel prisms. The mean of depth was calculated.

### 2.5. Scanning Electron Microscopy (SEM)

Eight of the sections examined in POLMI were prepared for SEM. After etching for 30 seconds with 30% phosphoric acid, they were carefully rinsed with deionized water and mounted on sample holders for the electron microscope. The sections used for the SEM analysis were coated with gold. The SEM examinations were carried out in a Philips SEM 515 at 20 kV (Philips, Eindhoven, The Netherlands) and in a field emission scanning electron microscope (Gemini IMB, Leo 1530, Oberkochen, Germany). The depths were measured from the surface of enamel to the bottom border of the structural change of enamel prisms.

### 2.6. X-Ray Microanalysis (XRMA)

#### 2.6.1. Pilotstudy

In order to investigate differences in the chemical content between different locations within the same tooth, nine teeth from nine individuals were analyzed. The mesio, buccal, and distal or mesio and distal sides of the enamel were analyzed with XRMA. The teeth were cut in sagittal or radial direction to sections of 100 *μ*m and coated with carbon by vapor deposition. The content of C, N, O, P, and Ca was analyzed in the enamel at 5 locations over a line from the enamel surface towards the enamel-dentin junction. The XRMA analysis was carried out in the same way as in the main study.

#### 2.6.2. Main Study

A section adjacent to those examined in POLMI (no = 18), was coated by vapor deposition with carbon for XRMA analysis in a LEO-1450VP (Zeiss, Oberkochen, Germany), at 12 keV with Quantax 200 (Bruker, AXS, Berlin, Germany) equipped with an XFlash 5030 detector and the ESPRIT Software for EDS (Bruker, AXS, Berlin, Germany). The XRMA analysis was performed at a magnification of 250 times, with a working distance of 25 mm. 

**Figure 1:**
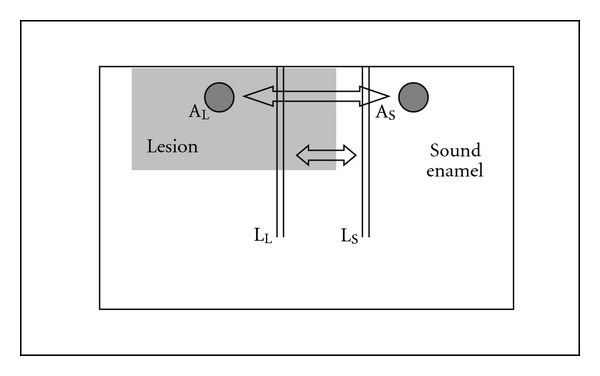
Schematic view of a specimen; the darker area shows the lesion and the white area indicate Sound enamel. Circles of the areas where measurements of XRMA were performed and the parallel vertical lines represent line scans of XRMA.

The elements analyzed were carbon, nitrogen, oxygen, phosphorous, and calcium. In order to compare the analyzed elements in demineralized enamel with sound enamel in each section, two line scans extending over 250 *μ*m, starting at the enamel surface, with 100 measuring points and a count rate of 4 kcps. The lines were made through the most coronally part of the lesion, towards the enamel-dentin junction following prism direction and through the adjacent coronally located sound enamel. The values along the two line scans were calculated and used for comparison between the lesion and the sound enamel in each section. Additional XRMA analyses were performed in predefined circles (diameter 30 *μ*m), 20 *μ*m below the surface in the lesion and in the neighboring coronally located sound enamel. These values reflect the relation of carbon, nitrogen, oxygen, phosphor, and calcium in the enamel and are used for comparison of the relative chemical content in weight percent (wt%) between teeth (Figure 1). All values are to be regarded as semiquantitative.

The depths of the lesions were measured in the SEM/XRMA image for each specimen, from the surface of the enamel to the bottom border of the structural change of the enamel prisms. One of the specimens was broken and the lesion was not found in the part of section that was still on the holder.

### 2.7. Statistical Analysis

To study differences between locations of the surface of individual tooth one-way ANOVA was performed in the pilot study. The same method was used when studying interindividual differences between chemical composition of the enamel surface.

Data were processed using SPSS software, version 15.0. Independent *t*-test was used to explore the groups when analyzing visual appearance and depth of lesion. One-way ANOVA was performed to compare differences between lesion and sound enamel. Spearman's rank correlation was utilized to analyze relationship between gray value and depth of lesion. The level of statistical significance was set to *P* > 0.05.

### 2.8. Inductive Analysis

An inductive analysis was performed to elucidate any relationship between the medical, histomorphological, and chemical variables. All data were compiled in an Excel spread sheet, where the values for the different variables were set in columns, each row representing one patient. The data were imported to the inductive analysis program XpertRule Miner (Attar Software, Lancashire, UK), where the columns represent *attributes* with different values (numeric or discrete). Before the analysis is performed, one of the discrete attributes is set as *outcome*. The results are presented in a hierarchic diagram (*knowledge tree*) in which the importance of every attribute in the inductive analysis is specified by its position in the knowledge tree. The higher in the tree, the more important for the outcome, and thus the tree shows how different attributes affect the outcome. In the induction process, a knowledge tree is generated by repeatedly splitting the given data set according to different attributes until terminal points (*leaves*) are reached.

The lesion depths (LD) measured in SEM/XRMA (*μ*m) were grouped into 3 groups: 0 < LD < 60; 60 ≤ LD < 100; LD ≥ 100. The 3 groups represent *discrete values* and were then used as *outcome* in the inductive analysis. The numerical and discrete data in the Excel file were used as attributes.

### 2.9. Ethical Considerations

This study was ethically conducted according to the Declaration of Helsinki and approved by the Ethical Committee at the Sahlgrenska Academy at the University of Gothenburg, Göteborg, Sweden, registered 432-08. The patients and their parents received oral and written information regarding the study. Participation was voluntary and written consent was given.

## 3. Results and Discussion

### 3.1. Pilotstudy

No differences were seen in the chemical content of C, N, O, P, or Ca within the individual sections between the different locations of surface of the tooth. When comparing the chemical content interindividually, differences in chemical content of N, O, P, and Ca were observed (*P* < 0.05) in the location closest to the enamel surface. However, variations in the chemical content between different individuals were larger compared with within the enamel of an individual.

The differences in chemical composition of the enamel in the pilot study, caused this analysis of lesion depth related to chemical properties of enamel. There are differences seen in the enamel composition as well as divergences in depth of demineralized lesions. Divergences in mineral loss, when demineralized, are also seen between the specimens. The demineralizing solution was from the same batch and did not differ in pH or buffer capacity, therefore differences in the depth of lesions cannot be explained by different properties of the acid.

### 3.2. Lesion Depth

#### 3.2.1. POLMI Examination

**Figure 2:**
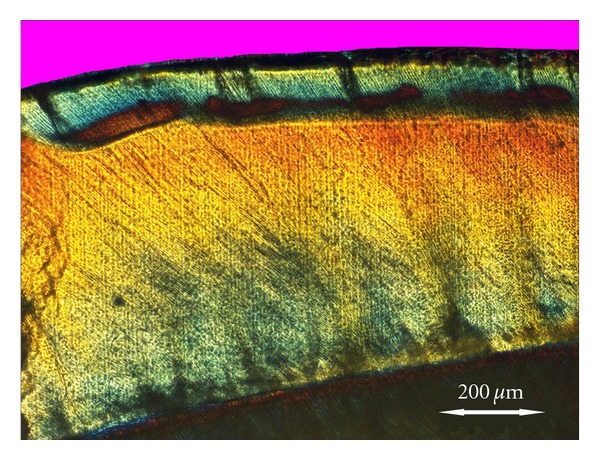
POLMI image, at 4x. The lesion is seen as a uniform demineralized zone, with a positive birefringent bulk below a negatively birefringent surface layer. At the lesion front, a zone with a higher degree of demineralization is seen.

In sound enamel, the enamel surface and the enamel zone below appeared negatively birefringent when examined dry in air. In the specimens, the depth of lesion varied (Table 1). Deeper lesions were seen as a fairly uniform demineralized zone, with a positive birefringence deep to a negatively birefringent surface layer. Shallow lesions were seen as a dark band just under the surface layer (Figure 2).

#### 3.2.2. Microradiography

A lesion was detectable in all microradiographs seen as a radio lucent area below a thin layer of radio opaque enamel surface. The lesion had a fairly consistent depth within each specimen; however, the depth showed variations between specimen (Table 1).

#### 3.2.3. SEM/XRMA

**Figure 3:**
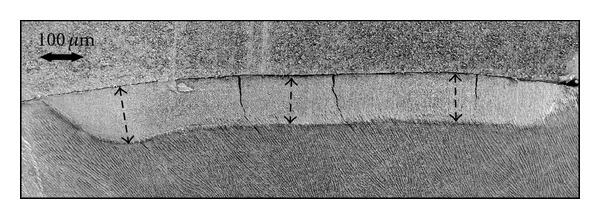
SEM image at 75x. The lesion appears as a well-defined area with distinct borders both in depth and width. Here is the lesion of lighter colour and having less structure compared to the sound enamel.

The lesion was seen as a homogenous change of structure with well-defined borders. The structure of the lesion appeared less patterned compared with sound enamel (Figure 3). The prisms were, however, still discernible, but no crystals were detectable in the lesion. Lesion depth measured in SEM and XRMA are seen in Table 1.

All the shallow lesions (0–60 *μ*m) were found among individuals older than 8 years, however, no statistical significant correlation was found (Table 1).

The appearance of the demineralized lesion in POLMI is in congruence with what has been in previous studies [12, 13].

The depth of the lesion was analyzed to the visual appearance of the surface after demineralization and indicates a more porous structure of the enamel. The depth of lesion was correlated to appearance of demineralized enamel. Though, different lesion depths do not correlate to divergences in thickness of the aprismatic surface layer of enamel [2]. Enormous variations of the chemical composition of the surface are reported, and this may influence the progress of demineralization [14].

The differences in lesion depth seen can be explained by the enamel surface having areas where the maturation process has not gone to completion. Therefore, small areas on the surface are more soluble [11]. This might result in different appearances of the surface on some of the teeth after acid exposure [15]. This explanation is in line with what has been shown here; the more affected surfaces had deeper lesions.

### 3.3. Description of the Lesions

#### 3.3.1. Surface Appearance

The exposed enamel surface differed in appearance between the specimens, 9 were chalky and rough, 5 were chalky and smooth, and 3 had a normal color with a smooth surface (Table 1).

#### 3.3.2. Microradiography

**Figure 4:**
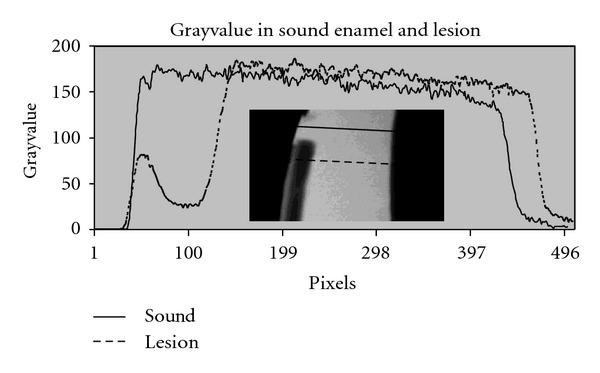
Image from a microradiography and graph of grayvalue. related to the image. Dotted line through the lesion and full line through the sound enamel.

A profile plot of the gray value across the enamel is shown in Figure 4.

The mean value of the Δgv was 69%, the gray value being lower in the lesion compared with the sound enamel.

#### 3.3.3. XRMA

There were noticeable differences in the content of carbon, nitrogen, calcium, and phosphorous in the lesions compared with sound enamel. The content of calcium was found to be lower in the lesion. Larger amounts of carbon and nitrogen parallel to less amounts of calcium and phosphorous were observed in the lesions compared with sound enamel in each individual on group level, nonparametric test ANOVA. The divergence in content of oxygen was less obvious and no significant differences between lesion and sound enamel were observed.

**Figure 5:**
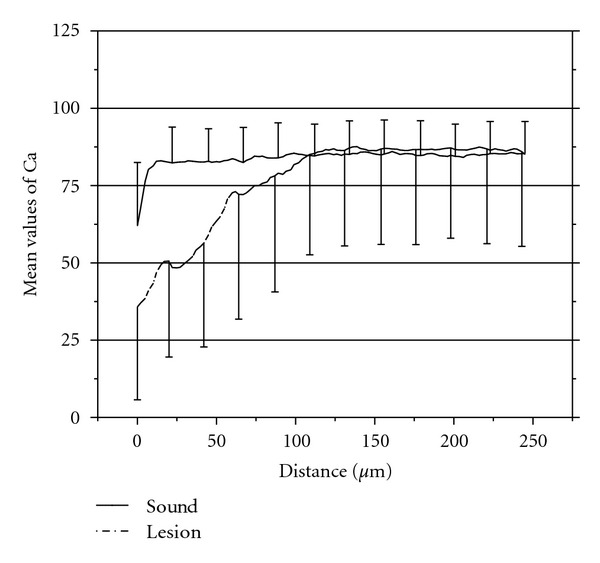
Graph of the mean values and standard deviations of wt% calcium in XRMA (linescans) in sound enamel and in lesion from the enamel surface till approximately 250 *μ*m in the enamel.

The content of calcium through a lesion made a clear change of content at the bottom border of the lesion. The content of calcium is illustrated in Figure 5.

The mean ratio of Ca : P was 2.1 in sound enamel and 1.7 in the lesion. The variances of semiquantitative wt% of measured elements are illustrated in Table 2.

The mean of ratio of Ca : P in sound enamel is in congruence of a study with larger material [6]. However, the significance of the ratio Ca : P is still not clear and it has been reported that the Ca : P ratio is stable irrespective of differences in the degree of mineralization [16], while others have left reasons open for discussion as to what the ratio actually represents [17].

There was a significant difference between sound enamel and the lesion, concerning the mean of the semiquantitative content of nitrogen. The content of nitrogen, on group level, was approximately twice as high in the lesion compared to the sound enamel (Table 2).

### 3.4. Correlations

The group of teeth showing chalky and rough surfaces also had the deepest lesions, compared to the group of teeth with a smooth surface. The visual appearance of the demineralized surface is correlated to the Δgv. The Δgv was lower when the surface of the demineralized lesion appeared chalky and rough. The depth of lesion is correlated to the Δgv in the lesion. The depth is greater when the Δgv loss is small.

The Δgv is smaller when the content of carbon in sound enamel is high. The gray value of sound enamel tended to be lower when the content of carbon was high in the sound enamel.

In this study, the specimen with the highest gray value in sound enamel, interpreted as the most well-mineralized enamel and not as porous, is showed to have lesions of more shallow depth. Specimens having a small Δgv, combined with deeper lesions, are to be interpreted as having a higher porosity of sound enamel. When sound enamel is porous, the gray value is comparably low. Due to the low gray value in sound enamel, the Δgv will also be smaller.

A specimen showing a small Δgv, a deep lesion and high content of carbon and also displaying a surface appearing chalky and rough after demineralization, is to be interpreted as the enamel having a large degree of porosity.

**Figure 6:**
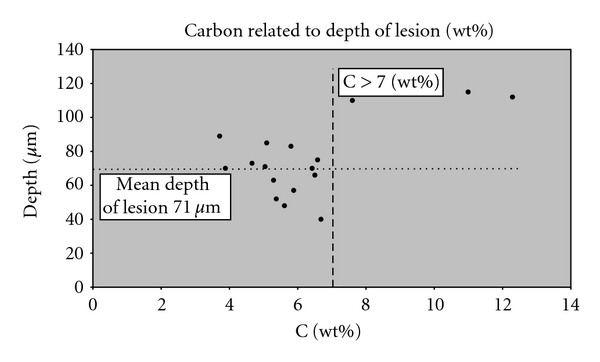
Plot of the wt% of carbon related to depth of lesion. Circle marks the specimen with wt% of carbon >7%. which also have deeper lesion.

Also, a correlation between the carbon content in sound enamel and the depth of the lesions was found. The carbon content in sound enamel was shown to be higher with deeper lesion depth. The specimens with a carbon wt% above 7% in sound enamel had deeper lesions (Figure 6).

This study has shown that the content of carbon in the sound enamel is relevant for the depth of a demineralized lesion. Sound enamel with a naturally higher content of carbon is creating pathways for diffusion and hereby explains the deeper lesions. The path taken by fluids through the enamel of extracted primary teeth is said to follow along the prism sheaths [18]. The incorporation of extraneous ions in hydroxyapatite crystals is changing the enamel's behavior towards acid. More carbonate in the enamel, interpreted as a substitute for phosphate and when existing in high doses, also substitutes the ion of hydroxyl, making the crystal less stable and facilitating the demineralization of enamel [9, 10].

Another interpretation is that sound enamel, with a high content of carbon, is reflecting a more porous enamel, where the porosities are infiltrated with cyanoacrylate from the glue. This process of infiltration is seen with other light viscous resins and is well known [19–23]. Therefore, the creation of deeper lesions in enamel, having less porous structure, is to be expected. This is in congruence with a greater lesion depth in the enamel of nonhuman primates compared to human enamel, where the enamel in primates has greater porosity [24]. Higher porosity due to the higher interprismatic fraction in primary teeth, compared to permanent teeth, has shown to be crucial to the depth of demineralized lesions [2].

There was no correlation in content of nitrogen in sound enamel and to the depth of the lesion.

The finding of higher content of nitrogen found in the demineralized enamel is in congruence to findings of the content of nitrogen in carious enamel being doubled compared to noncarious enamel on a per cent/weight basis [25]. Therefore, the finding of higher content of nitrogen in demineralized lesions seems correct.

### 3.5. Inductive Analysis

**Figure 7:**
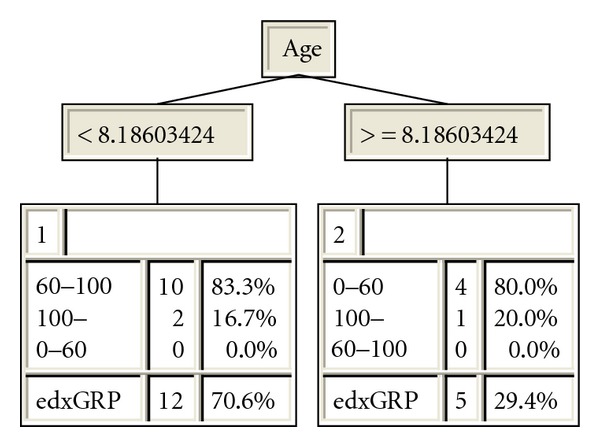
Knowledge tree generated in XpertRule Miner. At the top of the tree is the attribute “Age” located with the break points for the two end leaves with the group names, frequency within each group and percentage correctly classified examples. The last row shows the total frequency of examples and the overall correctly classified examples.

The inductive analysis revealed that the attribute *age* (age of the patient at the time for extraction) was the most important attribute for the outcome “*lesion depth*” measured in SEM/XRMA with a break point of 8.19 years of age (Figure 7). All other attributes thus were redundant. The lesion depth was between 60 and 100 *μ*m for 83% of the patients younger than 8.19 years, while 80% of the lesions was between 0 and 60 *μ*m among the patients older than 8.19 years.

Inductive analysis is a powerful complement to traditional statistical methods which has become more and more used in a variety of scientific fields since it may be applied both for quantitative and qualitative analysis [26–29]. The usage of sets of examples containing numeric as well as discrete variables makes it possible to structure information and find patterns and correlations which not always are discernable with traditional methods. This result was confirmed in the inductive analysis where 80% of the shallow lesions were derived from patients older than 8.19 years and 83% of the deeper lesions among patients younger than 8.91 years. The inductive analysis presents a hierarchic knowledge tree where the most important attribute always is located at the top node and only attributes relevant for the outcome is found in the knowledge tree. The break point of 8.19 years was not revealed in the statistical analysis of the material.

The hierarchic presentation of the result is easy to interpret and validate. The presented knowledge tree presents not only which attribute is the most important, the first node of the tree, but also which attributes are redundant for the outcome. However, it is important to realize that the presented tree does not represent an absolute truth why it has to be validated in relation to other scientific evidence or another set of examples. Therefore, an inductive analysis with its ability to use numeric as well as discrete attributes and its high explanatory value even with small sets of examples creates possibilities to look for patterns which else are difficult to discern. Inductive analysis or data mining is a technology which has been used in a huge range of fields and is a powerful complement to traditional statistical analytical methods [26–29]. The more shallow lesions seen in the older individuals in this study appear to be due to posteruptive incorporation of ions which is in congruence of findings in permanent teeth, where the progression of caries in vitro is slower in older individuals, especially after long-term exposure to fluorides [30]. Therefore, the result is a strong indicator of posteruptive effects of fluoride creating a more resistant enamel surface.

## 4. Discussion

In conclusion, the enamel responds to demineralization with different lesion depths and this correlates to the composition of the enamel. The lesion is deeper when the porosity of enamel is greater. There is a variety of chemical composition between the individual enamel analyzed.

The qualitative aspects of porosity to complement the quantitative degree of porosity would be interesting for future analyses regarding demineralization of the enamel. When studying the characterizations of enamel with variable caries risk, Gutierrez et al. found that the micropores between the hydroxyapatite crystals appeared to be laminar in the enamel in the high-risk caries group while the micropores were considered to be cylindrical in the low-risk caries group [31].

## Figures and Tables

**Table 1:** Mean of depth of lesions (in micrometer) in all specimens, measured in polarized light microscope (POLMI), microradiographs (MRG), SEM, and XRMA images. Appearance of lesions; chalky and rough surface, chalky and smooth surface, and normal color with a smooth surface. Age of the individuals in years when tooth was extracted.

	Depth in mm					
	POLMI	MRG	SEM	XRMA	Appearance	Age
1	74	71		57	Chalky and rough	13
2	110	46	58	48	Normal and smooth	13
3	87	82		83	Chalky and rough	5
4	112	49	47	70	Chalky and smooth	5
5	91	40		89	Chalky and rough	5
6	66	44	78	71	Chalky and rough	8
7	57	54	70	75	Chalky and rough	6
8	94	53	51	73	Normal and smooth	5
9	99	99	130	112	Chalky and rough	7
10	56	43		70	Chalky and smooth	7
11	38	25	31	40	Normal and smooth	13
12	74	72		110	Chalky and rough	17
13	87	37		66	Chalky and smooth	6
15	68	59		85	Chalky and smooth	6
16	77	38	46	63	Chalky and rough	3
17	81	73		115	Chalky and rough	6
18	109	75		52	Chalky and smooth	15

**Table 2:** Mean wt% of carbon, nitrogen, oxygen, phosphorous, and calcium in sound enamel and lesion.

	Chemical content in normal enamel and in lesion (weight%)									
	Carbon		Nitrogen		Oxygen		Phosphorous		Calcium	
	Sound	Lesion	Sound	Lesion	Sound	Lesion	Sound	Lesion	Sound	Lesion
Mean	6.21	23.33	2.04	5.49	41.02	40.72	15.90	11.34	34.82	19.13
Median	5.68	18.74	1.74	5.45	39.58	40.90	15.54	11.16	30.06	18.84
STD	2.13	11.95	1.02	1.67	3.68	6.30	1.30	01.30	4.18	7.97
